# Abundance of *Halyomorpha halys* (Hemiptera: Pentatomidae) and *Megacopta cribraria* (Hemiptera: Plataspidae) in Soybean in Areas with Few Previous Sightings in Tennessee

**DOI:** 10.3390/insects14030237

**Published:** 2023-02-27

**Authors:** Kaushalya G. Amarasekare, Richard H. Link

**Affiliations:** Department of Agricultural and Environmental Sciences, College of Agriculture, Tennessee State University, 3500, John A. Merritt Blvd., Nashville, TN 37209, USA

**Keywords:** brown marmorated stink bug (BMSB), kudzu bug, sweep sampling, insect monitoring, integrated pest management (IPM), seasonal abundance, lures

## Abstract

**Simple Summary:**

Invasive pests tend to move gradually to new areas from the initial location at which they were established or identified. *Halyomorpha halys* (Stål) and *Megacopta cribraria* (Fabricius) are two exotic invasive pests currently in the United States. *Halyomorpha halys* can damage various field crops such as soybean and corn and many species of fruits and vegetables, while *M*. *cribraria* attacks only soybean and the weed species kudzu. *Halyomorpha halys* and *M*. *cribraria* are a serious threat to soybeans, one of the major crops grown in Tennessee and in other southeastern states in the US. This research was focused on determining the establishment and seasonal abundances of *H*. *halys* and *M*. *cribraria* in soybeans in the central region of Tennessee in areas with few or no previous sightings of the two pests at the time of planning this study. Our results showed that both the *H*. *halys* and *M*. *cribraria* were established in the study locations, and the *H*. *halys* could become a serious pest during the pod-filling stage in September. The importance of monitoring these invasive pests in new areas with susceptible host plants to find their spread, establishment, and abundance is discussed.

**Abstract:**

*Halyomorpha halys* (Stål) and *Megacopta cribraria* (Fabricius) are two exotic invasive pests that have invaded the United States in recent years. *Halyomorpha halys* can damage various fruits, vegetables, and field crops, such as soybean and corn, while *Megacopta cribraria* only attacks soybean and kudzu, a weed species. They are currently found in southeastern states and threaten soybean and other crops grown in the region. This study evaluated the seasonal abundance of *H*. *halys* and *M*. *cribraria* in soybeans in 2016 and 2017 in two counties in the central region of Tennessee, where both species had either a few sightings or none that were recorded when this research was being planned. Lures and sweep sampling were used to monitor *H*. *halys*, and sweep sampling was used to monitor *M*. *cribraria*. *Halyomorpha halys* was first detected in samples in late July. Their numbers increased in early to mid-September, reached the economic threshold in late Sept, and then started to decline. *Megacopta cribraria* was first detected in mid to late July, increased their populations in September, but did not reach the economic threshold and declined mid-October. Our results showed the seasonal abundances of *H*. *halys* and *M*. *cribraria* and their establishment in the central region of Tennessee.

## 1. Introduction

*Halyomorpha halys* (Stål) (Hemiptera: Pentatomidae) is a polyphagous pest species that attacks more than a hundred fruits, vegetables, field crops, and ornamental plants in its native range of China, Japan, Korea, and Taiwan [1,2,3,4,5]. First found in Allentown, Pennsylvania, in 1998, it has a wide host range in the United States (US), including trees, shrubs, fruits, vegetables, and field crops, such as corn and soybean [1,4]. *Halyomorpha halys* has become a major pest in the Mid-Atlantic region, and there was a strong possibility that it would become a serious pest with regard to these commodities in Tennessee and other soybean-growing states in the southeastern US at the time of this study. Soybean, nursery production, and corn are three major crops in Tennessee, with soybeans being the most important [6,7]. It is also the state’s most important agricultural export crop. In 2014, Tennessee ranked 17 out of 31 soybean-producing states in the US [7]. *Halyomorpha halys* was first found in Tennessee in 2008 in Knox County in east Tennessee [8,9,10]. It gradually spread to other areas in the state at the time that this research was being planned in 2015 [10].

*Megacopta cribraria* (Fabricius) (Hemiptera: Plataspidae) is an exotic invasive pest from Asia [11,12,13]. It is a host-specific species, and the kudzu plant (*Pueraria* sp.) (family: Fabaceae, subfamily Faboideae), a noxious weed species, and soybeans are the primary reproductive hosts for this pest [11,12,13]. The kudzu plant is a common invasive weed in the southeastern US [11]. *Megacopta cribraria* was first discovered in the US in Georgia in 2009, and its distribution has rapidly grown to include the southeastern US and mid-south [11,12,13]. During the planning stage of this study, *M*. *cribraria* was expected to spread across the southern region of the US, particularly in areas where soybeans were grown and the kudzu plants were common [14]. Since invading the southeastern US, *M*. *cribraria* has become an economically damaging pest that can cause more than 60% yield loss [12,13]. The first report of *Megacopta cribraria* in Tennessee was in 2012 in the eastern region of the state [15,16,17], and it was subsequently reported on kudzu plants and soybeans from multiple counties in eastern and middle Tennessee [16,18]. The loss due to *M*. *cribraria* in Tennessee in 2016 was estimated to be over USD 1.5 million [19].

The objective of this study was to investigate the seasonal abundances of *H*. *halys* and *M*. *cribraria* in soybeans in two counties in the central region of Tennessee with either few or no previous recorded sightings of these pests, and to determine whether these pests could spread, establish and increase their populations sufficiently as to warrant management measures.

## 2. Materials and Methods

### 2.1. Field Site Selection and Experimental Design

Field experiments were conducted at the Tennessee State University’s Agricultural Research and Education Centers (TSU ARECs) in Nashville (Davidson County) [2016 and 2017] and Ashland City (Cheatham County) [2016], TN. An approximately 0.4 ha (96 m length × 45 m width) of land was used as the experimental plot to plant soybeans at each location. Land was prepared for planting in May 2016 and 2017, respectively. Seeds of soybean var. Hutchinson (Davidson Farmers Coop, Nashville, TN, USA) were directly sown into the soil using a planter/seeder at a row space of 0.8 m between rows, and 0.3 m apart within rows on 29 May 2016 (Nashville), 30 May 2016 (Ashland City) and 9 June 2017 (Nashville). When plants were approximately five wk old, each experimental plot was divided into four blocks using a randomized complete block design (RCBD) and assigned the treatments. *Halyomorpha halys* was monitored using sweep sampling and lures and *M*. *cribraria* was monitored using sweep sampling at weekly intervals. No insecticide treatments were applied to these plots in either location during the study period in both years.

#### *Halyomorpha halys* Monitoring

*Halyomorpha halys* enters crop fields from adjacent fields [20,21]. For this reason, two sweep sampling locations within each experimental block were used to monitor *H*. *halys*: (1) the border/edge of the soybean field adjacent to a fallow field, and (2) the center of the soybean field.

##### Sweep Sampling

Sweep samples were collected at each study location (at the border or the center of the field) within a block at the rate of 25 sweeps per block, using a 38.1 cm diameter sweep net (BioQuip Products, Inc., Rancho Dominguez, CA, USA). Twenty-five sweeps are generally used for pest monitoring in soybean [22]. The collected sweep samples from each block per location were placed in a labeled plastic bag and stored in a cooler until they were brought to the lab. Once in the lab, the insects were transferred to 500 mL plastic deli containers and the containers were placed in a freezer until they were evaluated. The sweep samples were examined under a stereomicroscope, and the number of *H*. *halys* nymphs and adults were counted and recorded. Antennal markings and wing characteristics were used to identify *H*. *halys* [1]. Weekly sweep samples were collected from July to October 2016 in Nashville and Ashland City and from July to October 2017 in Nashville.

##### Lures

A single lure treatment was used to monitor *H*. *halys* (Lure Treatment: Dead-Inn Combo Xtra lure [a combination lure of *H*. *halys* 2× lure + Harlequin bug lure]) (AgBio Inc., Westminster, CO, USA) in 2016, and two lure treatments (Lure treatment 1: Dead-Inn Combo Xtra lure; Lure treatment 2: HALHAL 25 lure [Alpha Scents Inc., West Linn, OR, USA]) in 2017. The second lure treatment (HALHAL 25) was added in 2017 to compare the efficiency of these two *H*. *halys* lures. *Halyomorpha halys* were placed in pyramid traps (AgBio Inc., Westminster, CO, USA) located in treatment plots at the rate of one trap/block/treatment (2016: one lure treatment (one trap/block) placed 24 m apart from block to block; 2017: two lure treatments (two traps/block) placed 12 m apart from block to block and between treatments within a block. The traps were placed along the same border, adjacent to a fallow land that was used for the border sweep sampling in July 2016 and 2017. Each lure treatment was attached to the interior wall of the collection bottle in the pyramid trap using general-purpose adhesive tape. An insect-killing strip (HERCON^®^ VAPORTAPETM II DDVP (10% dichlorvos [590 mg/strip] (2.5 cm × 10.2 cm) (Hercon Environmental, Emigsville, PA, USA) was secured to the interior wall of the collection bottle to kill the trapped insects before they escaped. The trapped insects were collected each week, placed into labeled plastic containers, and kept in a freezer until identification. The collected insects were examined under a stereomicroscope to identify and count the adult and immature *H*. *halys*. The lures and the insect-killing strips were replaced monthly.

### 2.2. Megacopta cribraria Monitoring

The abundance of *M*. *cribraria* was also monitored from the same sweep samples (25 sweeps/block) collected from the border and center sampling in Nashville and Ashland City in 2016 and Nashville in 2017 to monitor the abundance of *H*. *halys*. The collected sweep samples were examined under a stereomicroscope, and the number of immature and adult *M*. *cribraria* were counted and recorded. Morphological characteristics were used to identify immature and adult *M*. *cribraria* [11].

### 2.3. Statistical Analyses

A three-way repeated measures ANOVA (analysis of variance) for border and center sweep sampling was used to compare the location × treatment × date and year × treatment × date, and a two-way repeated measure ANOVA for lure treatments to compare the location × date, and year × date for the mean number of *H*. *halys* or *M*. *cribraria* in Ashland City and Nashville in 2016, and Nashville in 2016 and 2017, respectively (Statistical Analysis Software Proc Mixed Procedure) [23]. A three-way repeated measures ANOVA was used to compare the location × treatment × date to compare the efficiency of lure treatments over border sweep sampling for *H*. *halys* collected in Nashville and Ashland City in 2016, and a two-way repeated measures ANOVA was used to compare the treatment × date for the mean number of *H*. *halys* collected from the border sweep sampling and combined lure treatments (lure treatment 1 + lure treatment 2) in Nashville in 2017, respectively. A significance level of *p* ≤ 0.05 was used to compare the means using LSMEANS (least square means) for all experiments [23]. In the absence of interaction, if one of the main effects was not significant (*p* > 0.05), the data was pooled for that main effect and then analyzed accordingly. When both main effects were significant, one effect was evaluated on the levels of the other effect. If there was a significant interaction between the main effects, a simple means ANOVA was used to analyze the data for each level of one effect on the levels of the other effect.

#### Voucher Specimens

Voucher specimens of *H*. *halys* and *M*. *cribraria* were deposited in the arthropod collection at the TSU’s Department of Agricultural and Environmental Sciences, Fruit, Vegetable, and Field Crop Entomology Laboratory in Nashville, TN, USA.

## 3. Results

### 3.1. Halyomorpha halys

#### 3.1.1. Border and Center Sweep Sampling—Nashville and Ashland City in 2016

**Figure 1:**
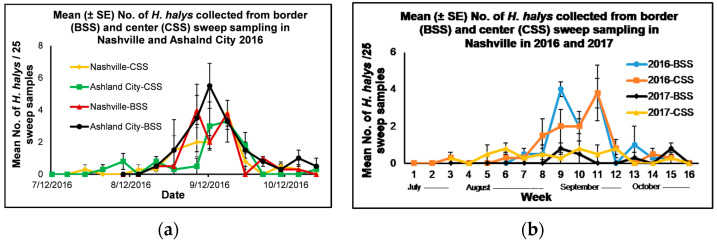
Mean (±SE) number of adult and immature *Halyomorpha halys* per 25 sweep samples/block collected from border and center sweep sampling in soybeans from (**a**) July to October 2016 conducted in Ashland City (Cheatham County) and Nashville (Davidson County), Tennessee (**b**) July to October 2016 and 2017 conducted in Nashville (Davidson County), Tennessee. Week 1: 7/12/2016 or 7/11/2017, week 2: 7/19/2016 or 7/18/2017, week 3: 7/26/2016 or 7/25/2017, week 4: 8/03/2016 or 8/02/2017, week 5: 8/10/2016 or 8/09/2017, week 6: 8/17/2016 or 8/16/2017, week 7: 8/24/2016 or 8/23/2017, week 8: 8/31/2016 or 8/30/2017, week 9: 9/07/2016 or 9/06/2017, week 10: 9/14/2016 or 9/13/2017, week 11: 9/21/2016 or 9/20/2017, week 12: 9/28/2016 or 9/27/2017, week 13: 10/05/2016 or 10/04/2017, week 14: 10/12/2016 or 10/11/2017, week 15: 10/19/2016 or 10/18/2017, and week 16: 10/26/2016 or 10/25/2017.

In 2016, the mean number of adult and immature *H*. *halys* collected from the border and center sweep sampling for Nashville was in the range of 0–3.8 for both methods, and 0–5.5 and 0–3.3, respectively, for Ashland City (Figure 1a). *Halyomorpha halys* started to appear in late July to early August and increased their numbers in mid-Sept. The mean number of adult and immature *H*. *halys* collected from the border sweep sampling in Ashland City in mid-September was within the threshold of 5–8 adult and immature stink bugs per 25 sweep samples for soybeans [24,25,26]. Their populations started to decline during the latter part of September. There was no significant interaction between location × treatment × date (F = 0.6, df = 11, 165, *p* = 0.83), location × treatment (F = 2.27, df = 1, 165, *p* = 0.13), location × date (F = 1.26, df = 15, 165, *p* = 0.23) and treatment × date (F = 1.67, df = 11, 165, *p* = 0.08) for the mean number of *H*. *halys* collected from border and center sweep sampling in Ashland City and Nashville in 2016. Out of the three main effects, the location effect was not significant, although the treatment and date effects were significant (F = 1.63, df = 1, 165, *p* = 0.05 and F = 3.82, df = 15, 165, *p* = <0.0001, respectively). When the data was analyzed with pooled locations, there was a significant interaction between treatment × date (F = 1.95, df = 11, 81, *p* = 0.04), and the two main effects, the treatment and date effects, were significant (F = 4.47, df = 1, 81, *p* = 0.04 and F = 13.23, df = 15, 81, *p* = <0.0001, respectively). When the treatment effect for each date was analyzed using a simple means one-way ANOVA (Proc Mixed) [23], there was a significant treatment effect between the mean number of adult and immature *H*. *halys* collected from the border and center sweep sampling on the weekly sampling date of 7 September 2016 (F = 8.33, df = 1, 3, *p* = 0.05). The mean number (±SE) of adult and immature *H*. *halys* collected from border sweep sampling (7.5 ± 1.9) was significantly higher than the mean number of *H*. *halys* collected from the center sweep sampling (2.5 ± 0.9) on 7 September 2016 (t = 2.89, df = 3, *p* = 0.05).

#### 3.1.2. Border and Center Sweep Sampling in Nashville in 2016 and 2017

The mean number of adult and immature *H*. *halys* collected from both border and center sweep sampling in Nashville was in the range of 0–3.8 in 2016 and 0–0.8 in 2017, respectively (Figure 1b). During both years, the mean number of *H*. *halys* collected from border and center sweep sampling was below the threshold of *H*. *halys* for 25 sweep samples for soybeans [24,25,26]. *Halyomorpha halys* started to appear in late July to early Aug, and the population started to increase in mid-September and then declined during the latter part of Sept. There was no significant interaction between year × treatment × date (F = 0.48, df = 11, 165, *p* = 0.92), treatment × date (F = 1.60, df = 13, 165, *p* = 0.09), and year × treatment (F = 1.29, df = 1, 165, *p* = 0.26) for the mean number of *H*. *halys* collected in weekly samples in 2016 and 2017 in Nashville, although there was a significant interaction between year × date (F = 7.20, df = 13, 165, *p* = < 0.0001). Out of the three main effects, the treatment effect was not significant, although the year and date effects were significant (F = 30.25, df = 1, 165, *p* = < 0.0001 and F = 8.64, df = 15, 165, *p* = < 0.0001, respectively). When the data with pooled treatments was analyzed, there was a significant interaction between year × date (F = 9.19, df = 13, 87, *p* = < 0.0001), and both main effects, the year and date, were significant (F = 36.35, df = 1, 87, *p* = < 0.0001 and F = 11.91, df = 15, 87, *p* = < 0.0001, respectively). A simple means one-way ANOVA was used to compare the mean number of *H*. *halys* collected from pooled sweep sampling in 2016 and 2017. There was a significant year effect for the mean number of *H*. *halys* collected from weekly sweep sampling between each week of the first three weeks of September in 2016 and the respective weeks in 2017 (7 September 2016 and 6 September 2017, 14 September 2016 and 13 September 2017, and 21 September 2016 and 20 September 2017) (F = 13.64, df = 1, 3, *p* = 0.03, F = 33.0, df = 1, 3, *p* = 0.01 and F = 36.75, df = 1, 3, *p* = 0.01, respectively). A significantly higher mean number of *H*. *halys* was collected from the sweep sampling in Nashville during the first three weeks of weekly collection dates in September in 2016 (7, 14, and 21 September) (6.0 ± 1.3, 4.0 ± 0.9 and 7.5 ± 1.0, respectively) than the first three weeks of weekly collection dates in September in 2017 (6, 13, and 20 September) (1.0 ± 0.4, 1.3 ± 0.9 and 0.5 ± 0.5, respectively) (t = 3.69, df = 3, *p* = 0.03, t = 5.74, df = 3, *p* = 0.01 and t = 6.06, df = 3, *p* = 0.01, respectively).

#### 3.1.3. Lures in Ashland City and Nashville in 2016

**Figure 2:**
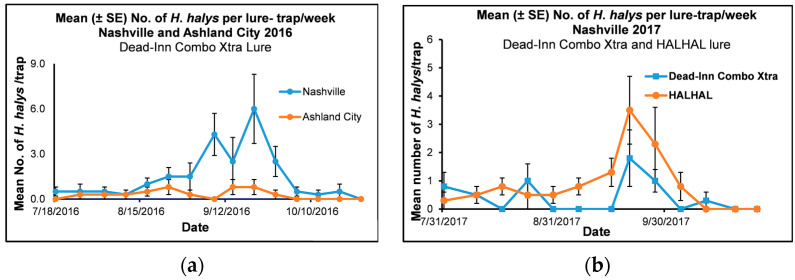
Mean (±SE) number of adult and immature *Halyomorpha halys* collected per trap/week in soybeans from (**a**) July to October 2016 in Nashville (Davidson County) and Ashland City (Cheatham County), Tennessee using a Dead-Inn Combo Xtra lure (combined Dead-Inn *H*. *halys* and *Harlequin* bug lures) with *H*. *halys* pyramid traps (**b**) July to October 2017 conducted in Nashville (Davidson County), Tennessee using two *H*. *halys* lures (Dead-Inn Combo Xtra [combined Dead-Inn *H*. *halys* and *Harlequin* bug lures] and HALHAL 25) with *H*. *halys* pyramid traps.

The mean number of adult and immature *H*. *halys* collected from lure treatments in Ashland City and Nashville in 2016 was in the range of 0–0.8 and 0–6, respectively (Figure 2a). *Halyomorpha halys* started to appear in late July to early August in Ashland City, and the population remained low throughout the study period. In Nashville, *H*. *halys* started to appear in late July, and the population started to increase in mid-to late September and declined in early October. There was a significant interaction between location × date for *H*. *halys* collected from lures in Ashland City and Nashville in 2016 (F = 2.91, df = 14, 87, *p* = 0.001), and both the main effects and the location and date effects were significant (F = 25.94, df = 1, 87, *p* = <0.0001 and F = 4.04, df = 14, 87, *p* = 0.001, respectively). We used a simple means one-way ANOVA to analyze the mean number of *H*. *halys* collected on each date by location. The mean number of *H*. *halys* collected from the lure treatment was significantly higher in Nashville (4.3 ± 1.4) than the mean number of *H*. *halys* collected from lures in Ashland City (0.0 ± 0.0) (F = 9.53, df = 1, 3 *p* = 0.05) on 7 September 2016.

#### 3.1.4. Lures in Nashville 2017

The mean number of adult and immature *H*. *halys* collected from Dead-Inn Combo Xtra and HALHAL 25 lure treatments were in the range of 0–1.8 and 0–3.5, respectively, in Nashville in 2017 (Figure 2b). *Halyomorpha halys* started to appear in late July, and the population started to increase in mid-to late September and declined in early Oct. There was no significant interaction between the date the traps were collected × lure treatments in Nashville in 2017 (F = 1.31, df = 12, 75, *p* = 0.23), although both main effects, the date (F = 5.45, df = 12, 75, *p* < 0.0001) and treatment (F = 6.49, df = 1, 75, *p* = 0.01) effects were significant. The results of the treatment comparison for each date showed that the mean number (± SE) of *H*. *halys* collected from the traps with HALHAL lure (1.3 ± 0.5, 3.5 ± 1.2, and 2.3 ± 1.3) was significantly higher than the mean number *H*. *halys* caught in the traps with Dead-Inn Combo Xtra lure (0.0 ± 0.0, 1.8 ± 1.0, and 1.0 ± 0.4) on September 13, 20, and 27, 2017, respectively (t = −2.00, df = 75, *p* = 0.05, t = −2.80, df = 75, *p* = 0.01 and t = −2.00, df = 75, *p* = 0.05, respectively) (LSMEANS) (PROC MIXED).

#### 3.1.5. Comparison of Lure Treatment and Border Sweep Sampling in Nashville and Ashland City in 2016

**Figure 3:**
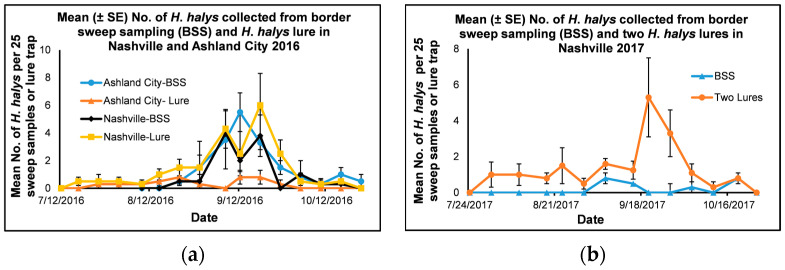
Mean (±SE) number of adult and immature *Halyomorpha halys* per 25 sweep samples/block collected from (**a**) border sweep sampling (BSS) or *H*. *halys* lure (Dead-Inn Combo Xtra lure) in soybeans from July to October 2016 conducted in Ashland City (Cheatham County) and Nashville (Davidson County), Tennessee (**b**) border sweep sampling (BSS) or both *H*. *halys* lures (Dead-Inn Combo Xtra and HALHAL 25) in soybeans from July to October 2017 conducted in Nashville (Davidson County), Tennessee.

To find the effectiveness of the lure treatment and border sweep sampling, we compared the mean number of adult and immature *H*. *halys* collected from the lure treatment with the mean number of *H*. *halys* collected from the border sweep sampling in Ashland City and Nashville in 2016 (Figure 3a). There is no established threshold for *H*. *halys* for lure treatments for soybeans. The threshold of *H*. *halys* in apples is ten adult and immature stink bugs per weekly trap collection [27]. Although we cannot compare the threshold of apples for soybeans, our assumption was that *H*. *halys* lure treatment would catch more stink bugs than the border sweep samples. The mean number of *H*. *halys* collected from the border sweep sampling and lure treatment was in the range of 0–4, 0–6, and 0–5.5, 0–0.8, respectively, for Nashville and Ashland City. There was no significant interaction between location × treatment × date (F = 1.60, df = 11, 141, *p* = 0.10) and treatment × date (F = 1.31, df = 11, 141, *p* = 0.22) for the mean number of *H*. *halys* collected from lures and border sweep sampling in Ashland City and Nashville in 2016. There was a significant interaction between the location × treatment (F = 19.39, df = 1, 141, *p* = < 0.0001) and location × date (F = 1.98, df = 11, 141, *p* = 0.04), and the main effects location (F = 4.54, df = 1, 141, *p* = 0.04) and date were significant (F = 9.66, df = 11, 141, *p* = < 0.0001).

When the treatment and date effect were analyzed for each location, there was a significant interaction between treatment × date (F = 2.72, df = 11, 69, *p* = < 0.0001) for *H*. *halys* collected from lure treatment and border sweep sampling in Ashland City in 2016, and both main effects, the treatment (F = 19.19, df = 1, 69, *p* = < 0.0001) and date (F = 3.63, df = 11, 69, *p* = 0.0004) were significant. When the treatment effect was analyzed for each date using a simple means one-way ANOVA, there was a significant treatment effect for the mean number of *H*. *halys* collected from the border sweep sampling and lure traps on August 31, 2016 (F = 25.0, df = 1, 3, *p* = 0.02). The mean number of *H*. *halys* collected from the border sweep sampling (1.5 ± 0.3) was significantly higher than the mean number of *H*. *halys* collected from the lure traps (0.5 ± 0.3) (t = 5.00, df = 3, *p* = 0.02) on 31 August 2016.

There was no interaction between treatment × date for the mean number of *H*. *halys* collected from weekly border sweep sampling and lure treatment in Nashville in 2016 (F = 0.65, df = 11, 69, *p* = 0.78). However, both main effects, the treatment and date effects were significant (F = 4.76, df = 1, 69, *p* = 0.03 and F = 7.98, df = 11, 69, *p* = <0.0001, respectively). When the mean number of *H*. *halys* collected from the border sweep sampling and lure treatment for each date was compared using a simple means one-way ANOVA, the mean number of *H*. *halys* collected from the lure treatment was significantly higher than the mean number of *H*. *halys* collected from the border sweep sampling on September 21 (6.0 ± 2.3 and 3.8 ± 1.5) and September 28 (2.5 ± 1.0 and 0.0 ± 0.0) 2016 (t = −2.00, df = 69, *p* = 0.05 and t = 2.22, df = 69, *p* = 0.03, respectively).

#### 3.1.6. Comparison of Combined Lure Treatments and Border Sweep Sampling in Nashville in 2017

To compare the effectiveness of lure treatments and border sweep sampling in Nashville in 2017, the mean numbers of *H*. *halys* collected from both lure treatments (Dead-Inn Combo Extra lure and HALHAL 25 lure) were combined and compared with the mean number of *H*. *halys* collected from the border sweep sampling (Figure 3b). The mean number of *H*. *halys* collected from the border sweep sampling and the combined lure treatments were in the range of 0–0.8 and 0–5.3, respectively. There was a significant interaction between treatment × date for the mean number of *H*. *halys* collected from the weekly combined lure treatments and border sweep sampling (F = 3.71, df = 12, 75, *p* = 0.0002). Both main effects and the treatment and date were significant (F = 22.72, df = 1, 75, *p* = < 0.0001 and F = 2.99, df = 12, 75, *p* = 0.002, respectively). When the treatments for each collection date were analyzed using a simple means one-way ANOVA, there was a significant treatment effect between the mean number of *H*. *halys* collected from the combined lure treatment and border sweep sampling on 20 September (F = 5.63, df = 1, 3, *p* = 0.05) and 27 September 2017 (F = 6.76, df = 1, 3, *p* = 0.04). The mean number of *H*. *halys* collected from the lure treatments was significantly higher than the mean number of *H*. *halys* collected from the border sweep sampling on 20 September (5.3 ± 2.2 and 0.0 ± 0.0, t = −2.37, df = 3, *p* = 0.05) and 27 September (3.3 ± 1.3 and 0.0 ± 0.0, t = −2.60, df = 3, *p* = 0.04), respectively.

### 3.2. Megacopta cribraria

#### 3.2.1. Border and Center Sweep Sampling in Ashland City and Nashville in 2016

**Figure 4:**
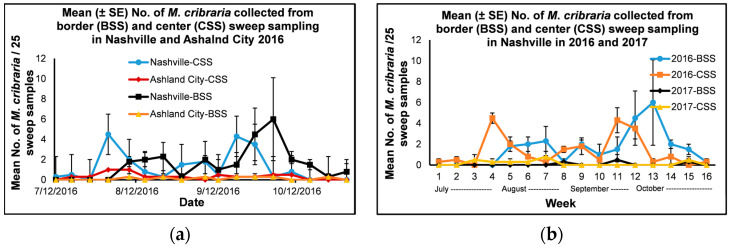
Mean (± SE) number of nymphs and immature *Megacopta cribraria* per 25 sweep samples collected from border and center sweep sampling in soybeans from (**a**) July to October 2016 conducted in Ashland City (Cheatham County) and Nashville (Davidson County), Tennessee (**b**) July to October 2016 and 2017 in Nashville (Davidson County), Tennessee. Week 1: 7/12/2016 or 7/11/2017, week 2: 7/19/2016 or 7/18/2017, week 3: 7/26/2016 or 7/25/2017, week 4: 8/03/2016 or 8/02/2017, week 5: 8/10/2016 or 8/09/2017, week 6: 8/17/2016 or 8/16/2017, week 7: 8/24/2016 or 8/23/2017, week 8: 8/31/2016 or 8/30/2017, week 9: 9/07/2016 or 9/06/2017, week 10: 9/14/2016 or 9/13/2017, week 11: 9/21/2016 or 9/20/2017, week 12: 9/28/2016 or 9/27/2017, week 13: 10/05/2016 or 10/04/2017, week 14: 10/12/2016 or 10/11/2017, week 15: 10/19/2016 or 10/18/2017, and week 16: 10/26/2016 or 10/25/2017.

Only a few *M*. *cribraria* immatures and adults were collected from the sweep sampling in Nashville and Ashland City in 2016, and thus the number of immatures and adults were combined and used in the analyses. The mean number of adult and immature *M*. *cribraria* collected from both border and center sweep sampling in 2016 was within the range of 0–0.3, 0–1.0 and 0–6.0, 0–4.5 for Ashland City and Nashville, respectively (Figure 4a). The numbers collected were below the threshold level of 25 immature *M*. *cribraria* per 25 sweep samples throughout the study period [26,28]. *Megacopta cribraria* started to appear in mid to late July and increased their numbers in late September to early Oct. Their populations started to decline in mid-October. There was no significant interaction between location × treatment × date (F = 1.7, df = 11, 165, *p* = 0.08) and treatment × date (F = 1.62, df = 11, 165, *p* = 0.1) for the mean number of *M*. *cribraria* collected from border and center sweep sampling in Ashland City and Nashville in 2016, although there was a significant interaction between location × treatment (F = 4.10, df = 1, 165, *p* = 0.04) and location × date (F = 1.76, df = 15, 165, *p* = 0.04). Out of the three main effects, the treatment effect was not significant, although the location and date effects were significant (F = 43.54, df = 1, 165, *p* = < 0.0001 and F = 2.63, df = 15, 165, *p* = 0.001, respectively). When the data with pooled treatments was analyzed there was a significant interaction between location × date (F = 1.91, df = 15, 93, *p* = 0.03) and the two main effects, the location and date effects, were significant (F = 38.54, df = 1, 93, *p* = < 0.0001 and F = 2.60, df = 15, 93, *p* = 0.003, respectively). When the location effect was analyzed for each date for the mean number of *M*. *cribraria* collected from sweep sampling in Nashville and Ashland City in 2016 using a simple means one-way ANOVA, there was a significant location effect on 3 and 10 August, 7 and 21 September, and 12 October 2016 (F = 21.0, df = 1, 3, *p* = 0.02; F = 15.0, df = 1, 3, *p* = 0.03; F = 10.89, df = 1, 3, *p* = 0.05; F = 57.52, df = 1, 3, *p* = 0.01; and F = 12.79, df = 1, 3, *p* = 0.04, respectively). The mean number (± SE) of *M*. *cribraria* collected from sweep sampling in Nashville was significantly higher than the mean number of *M*. *cribraria* collected from the sweep sampling in Ashland City on 3 and 10 August, 7 and 21 September, and 12 October 2016 (3 August: 4.5 ± 0.5 and 1.0 ± 0.6 [t = −4.58, df = 3, *p* = 0.02]; 10 August: 3.8 ± 0.5 and 1.3 ± 0.5 [t = −3.87, df = 3, *p* = 0.03]; 7 September: 3.8 ± 1.0 and 0.3 ± 0.3 [t = −3.30, df = 3, *p* = 0.05]; 21 September: 5.8 ± 0.6 and 0.5 ± 0.3 [t = −7.58, df = 3, *p* = 0.01]; and 12 October: 2.8 ± 0.9 and 0.5 ± 0.3 [t = −3.58, df = 3, *p* = 0.04], Nashville and Ashland City, respectively).

#### 3.2.2. Border and Center Sweep Sampling in Nashville in 2016 and 2017

Only a few immature *M*. *cribraria* were collected from the sweep sampling in Nashville in 2016 and 2017, and thus the number of immature and adult *M*. *cribraria* were combined and used in the analyses. The mean number of immature and adult *M*. *cribraria* collected from the border and center sweep sampling in Nashville in 2016 and 2017 was within the range of 0–6.0, 0–4.5, and 0–0.5, 0–0.8, respectively (Figure 4b). The numbers collected were below the threshold level of 25 adult and immature *M*. *cribraria* per 25 sweep samples throughout the study period for both years [26,28]. The numbers collected were low for both border and center sweep sampling in Nashville in 2017. In Nashville in 2016, *M*. *cribraria* started to appear in mid-to late July and increased their numbers in late September to early October. Their populations started to decline in mid-October. There was a significant interaction between year × treatment × date (F = 1.91, df = 11, 165, *p* = 0.04) and year × date (F = 2.52, df = 13, 165, *p* = 0.004), although the interaction between treatment × date (F = 1.24, df = 13, 165, *p* = 0.26) and year × treatment (F = 3.29 df = 13, 165, *p* = 0.07) was not significant. Out of the three main effects, the treatment effect was not significant, although the year and date effects were significant (F = 54.98, df = 1, 165, *p* = < 0.0001 and F = 2.14, df = 15, 165, *p* = 0.01, respectively). When the pooled treatment data were analyzed, there was a significant interaction between year × date (F = 2.17, df = 13, 87, *p* = 0.02), and both main effects and year and date were significant (F = 48.16, df = 1, 87, *p* = < 0.0001 and F = 2.39, df = 15, 87, *p* = 0.01, respectively). n A simple means one-way ANOVA was used to compare the year effect for the weekly mean number of *M*. *cribraria* collected from sweep sampling in 2016 and 2017. There was a significant year effect for the mean number of *M*. *cribraria* collected from weekly sweep sampling on 3 August 2016 and 2 August 2017, 10 August 2016 and 9 August 2017, 7 September 2016 and 6 September 2017, 21 September 2016 and 20 September 2017, and 12 October 2016 and 11 October 2017 (F = 57.8, df = 1, 3, *p* = 0.01, F = 42.0, df = 1, 3, *p* = 0.01, F = 13.24, df = 1, 3, *p* = 0.04, F = 120.27, df = 1, 3, *p* = 0.002 and F = 10.37, df = 1, 3, *p* = 0.05, respectively). A significantly higher number of *M*. *cribraria* was collected from the weekly sweep sampling on 3 and 10 August, 7 and 21 September, and 12 October 2016 (4.5 ± 0.5, 3.8 ± 0.5, 3.8 ± 1.0, 5.8 ± 0.6 and 2.8 ± 0.9, respectively) than the respective weekly sampling days on 2 and 9 August, 6 and 20 September, and 11 October 2017 (0.3 ± 0.3, 0.3 ± 0.3, 0.0 ± 0.0, 0.5 ± 0.5 and 0.0 ± 0.0, respectively) (t = 7.60, df = 3, *p* = 0.01, t = 6.48, df = 3, *p* = 0.01, t = 3.64, df = 3, *p* = 0.04, t = 10.97, df = 3, *p* = 0.002, and t = 3.22, df = 3, *p* = 0.05, respectively).

## 4. Discussion

This research focused on determining the establishment and seasonal abundance of *H*. *halys* and *M*. *cribraria* in soybeans in 2016 and 2017 in two locations in the central region of Tennessee, with few or no previous sightings of the pests at the time of the planning of this study in 2015. Adult and immature *H*. *halys* were monitored using traditional sweep sampling and commercially available semiochemical lures for *H*. *halys,* and only sweep samples were used to monitor *M*. *cribraria*. Both species were present in study locations in both years. However, the abundance of *H*. *halys* was low throughout the study period except during September, when the population was within the threshold of 5–8 adults and immatures per 25 sweeps (~3–5 adults and immatures for 15 sweeps) for soybeans [24,25,26], which may warrant management measures. In contrast, the population of *M*. *cribraria* was below the threshold of 25 immatures per 25 sweeps for soybeans [26,28] throughout the study period.

There was a significant location effect for *M*. *cribraria* sweep sampling in 2016 and a significant year effect for both *H*. *halys* and *M*. *cribraria* in 2016 and 2017. The border sweep sampling performed better than the center sweep sampling in collecting *H*. *halys* in 2016. This may be because *H*. *halys* enters crop fields from adjacent fields and/or field borders [20,21]. The semiochemical lures performed better than the border sweep sampling in locations with a higher population of *H*. *halys* than in a lower population. The performance of *H*. *halys* lure treatments varied with the type of lure used. Out of the two lures tested in 2017, the HALHAL lure performed better than the Combo Xtra lure. There is no established threshold for the mean number of adult and immature *H*. *halys* collected weekly per trap per lure treatment for the soybeans; thus, we could not compare our results with a threshold.

In this study, *H*. *halys* started to appear in late July to early August, and their populations remained low until early September. The peak population was in mid-September during the pod-forming and seed-filling stages of soybeans. Previous research shows that the initial appearance of *H*. *halys* varies according to location and crop type [29,30]. Studies conducted in Maryland showed that *H*. *halys* appeared earlier in the season in the field crops such as corn (mid-July) and vegetables such as eggplant (early July) and bell pepper (mid-July) than in okra (late July), beans (early August), and tomato (late August) [30]. These studies also showed that there were two generations of *H*. *halys* in crops such as bell pepper and eggplant [30].

Tennessee has two generations of *M*. *cribraria*; its first generation develops on kudzu plants, while the second generation develops on kudzu and soybeans [17]. In this study, we observed one generation of *M*. *cribraria* on soybeans in both experimental locations. Considering the time of planting and its appearance on treatment plots, the samples of *M*. *cribraria* collected from the study locations could be from the second generation of *M*. *cribraria*. The soybeans for this study were planted in late May and early June. Results of a study conducted in Georgia on finding the planting date and injury of *M*. *cribraria* to soybeans show that there was only one generation of *M*. *cribraria* when planting soybeans in June or July, and the late-planting soybeans had less damage from *M*. *cribraria* than early-planting soybeans [31]. Planting soybeans before the end of May or early June helps mitigate freeze damage to young soybean plants. The last freeze in the study locations in central Tennessee is generally expected in mid-May.

After initial detection, it takes five or more years for *H*. *halys* to develop as a significant pest in a location, and when established, it can become a highly destructive pest [3]. After the initial detection of *H*. *halys* in the eastern region of Tennessee in 2008, it gradually spread to other areas in the state. These studies were conducted approximately eight years after the initial detection of *H*. *halys* in east Tennessee. Our results show that although *H*. *halys* has not yet become a destructive pest in the study locations in the central region of Tennessee, its population could increase in September during the pod-forming and seed-filling stages of soybeans and subsequently damage the crop.

It is possible that the high environmental temperatures observed during the summer months in Tennessee in 2016 and 2017 negatively affected the development of *H*. *halys* and contributed to its low abundance exhibited in this study. Temperature plays a vital role in the development, reproduction, and survival of *H*. *halys* in a region [29]. Studies conducted in the mid-Atlantic region to find the abundance of *H*. *halys* in soybeans showed that this invasive pest might not be found in high numbers in crop fields with an average monthly temperature higher than 23.5 °C [29]. The average monthly temperature recorded for both experimental locations of this study in June, July, August, September, and October was 26.3, 28.1, 27.5, 24.4, 19.8 °C, and 24.6, 27.4, 25.4, 22.1, 17.0 °C in 2016 and 2017, respectively [32]. Our results show a trend between *H*. *halys* abundance and monthly average temperature similar to the studies conducted in mid-Atlantic states [29].

*Beauveria bassiana* (Balsamo) Vuillemin (Hypocreales: Cordycipitaceae), a Deuteromycete and a naturally occurring entomopathogenic fungus, was found in *M*. *cribraria* in east Tennessee in 2015 while studying its life history, seasonality, and phenology in the field [17]. That study showed a reduction in the immature *M*. *cribraria* populations due to *B*. *bassiana* infestations, which led to a low adult population [17]. The activity of *B*. *bassiana* on *M*. *cribraria* was not assessed in this current study; thus, it is not possible to confirm that the low population densities of *M*. *cribraria* observed in this study could be due to any infestations of immature *M*. *cribraria* caused by *B*. *bassiana*.

This study presents findings on the seasonal abundance of *H*. *halys* and *M*. *cribraria* in new locations with either few previously recorded sightings or no sightings in the central region of Tennessee. It provides insight into the pest status of these two exotic invasive species in soybeans in the state. According to our results, *H*. *halys* could become a serious pest of soybeans during the pod-filling stage in September, depending on the location and year, and populations of *M*. *cribraria* may not reach economically damaging levels in soybeans in central Tennessee.

## Data Availability

Data will be available for reasonable requests addressed to the corresponding author.

## References

[1] Hoebeke E.R., Carter M.E. (2003). *Halyomorpha halys* (Stal) (Heteroptera: Pentatomidae): A polyphagous plant pest from Asia newly detected in North America. Proc. Entomol. Soc. Wash..

[2] Leskey T.C., Hamilton G.C., Nielsen A.L., Polk D.F., Rodriguez-Saona C., Bergh J.C., Herbert D.A., Kuhar T.P., Pfeiffer D., Dively G.P. (2012). Pest status of the brown marmorated stink bug, *Halyomorpha halys* in the USA. Outlooks Pest Manag..

[3] Lee D.H., Short B.D., Joseph S.V., Bergh J.C., Leskey T.C. (2013). Review of the biology, ecology, and management of *Halyomorpha halys* (Hemiptera: Pentatomidae) in China, Japan, and the Republic of Korea. J. Econ. Entomol..

[4] Rice K.B., Bergh C.J., Bergmann E.J., Biddinger D.J., Dieckhoff C., Dively G., Fraser H., Gariepy T., Hamilton G., Haye T. (2014). Biology, ecology, and management of brown marmorated stink bug (Hemiptera: Pentatomidae). J. Integr. Pest Manag..

[5] Weber D.C., Leskey T.C., Walsh G.C., Khrimian A. (2014). Synergy of aggregation pheromone with methyl (E,E,Z)-2,4,6-decatrienoate in attraction of *Halyomorpha halys* (Hemiptera: Pentatomidae). J. Econ. Entomol..

[6] IPM Data (2006). Crop Profile for Soybeans in Tennessee. The National IPM Database..

[7] USDA-NASS (2015). United States Department of Agriculture-National Agricultural Statistics Service Crop Production Summary 2014. https://www.usda.gov/nass/PUBS/TODAYRPT/cropan15.pdf.

[8] Jones J.R., Lambdin P.L. (2009). New County and state record for Tennessee of an exotic pest, *Halyomorpha halys* (Hemiptera: Pentatomidae) with potential economic and ecological implications. Fla. Entomol..

[9] Addesso K., Oliver J. (2012). Brown Marmorated Stink Bug.

[10] Britt K., Standish C., Grant J., Vail K. (2018). Managing Brown Marmorated Stink Bug in and Around Homes.

[11] Eger J.E., Ames L.M., Suiter D.R., Jenkins T.M., Rider D.A., Halbert S.E. (2010). 0121. Halbert. Occurrence of the old-world bug Megacopta cribraria (Fabricius) (Heteroptera: Plataspidae) in Georgia: A serious home invader and potential legume pest. Insecta Mundi.

[12] Seiter N.J., Greene J.K., Reay-Jones F.P. (2013). Reduction of soybean yield components by *Megacopta cribraria* (Hemiptera: Plataspidae). J. Econ. Entomol..

[13] Seiter N.J., Greene J.K., Reay-Jones F.P., Roberts P.M., All J.N. (2015). Insecticidal control of *Megacopta cribraria* (Hemiptera: Plataspidae) in soybeans. J. Entomol. Sci..

[14] Ruberson J.R., Takasu K., David Buntin G., Eger J.E., Gardner W.A., Greene J.K., Jenkins T.M., Jones W.A., Olson D.M., Roberts P.M. (2013). From Asian curiosity to eruptive American pest: *Megacopta cribraria* (Hemiptera: Plataspidae) and prospects for its biological control. Appl. Entomol. Zool..

[15] Musser F.R., Catchot A.L., Davis J.A., Herbert D.A., Lorenz G.M., Reed T., Reisig D.D., Stewart S.D. (2013). 2012 Soybean insect losses in the Southern U.S. Midsouth Entomol..

[16] Addesso K., Oliver J. (2016). Kudzu Bug.

[17] Britt K.C. (2016). An Ecological Study of the Kudzu Bug in East Tennessee: Life History, Seasonality, and Phenology. Master’s Thesis.

[18] Kudzubug (2016). Org. Kudzu Bug. Bugwood Center for Invasive Species and Ecosystem Health.

[19] Musser F., Catchot A., Davis J., Lorenz G., Reed T., Reisig D., Stewart S., Taylor S. (2017). 2016 Soybean insect losses in the Southern U.S. Midsouth Entomol..

[20] Aigner B.L., Kuhar T.P., Herbert D.A., Brewster C.C., Hogue J.W., Aigner J.D. (2017). Brown marmorated stink bug (Hemiptera: Pentatomidae) infestations in tree borders and subsequent patterns of abundance in soybean fields. J. Econ. Entomol..

[21] Leskey T.C., Nielsen A.L. (2018). Impact of the invasive brown marmorated stink bug in North America and Europe: History, biology, ecology, and management. Annu. Rev. Entomol..

[22] McPherson R.M., Smith J., Allen W.A. (1982). Incidence of arthropod predators in different soybean cropping systems. Environ. Entomol..

[23] SAS Institute (2013). Statistical Analysis Software (SAS) User’s Guide. Version 9.4.

[24] Tooker J. (2012). Brown marmorated stink bug as a pest of corn and soybeans. Entomological Notes.

[25] Virginia Cooperative Extension (2015). Brown Marmorated Stink Bug.

[26] Greene J.K. (2020). Soybean insect control. 2020 South Carolina Pest Management Handbook.

[27] Short B.D., Khrimian A., Leskey T.C. (2017). Pheromone-based decision support tools for management of *Halyomorpha halys* in apple orchards: Development of a trap-based treatment threshold. J. Pest Sci..

[28] Stewart S.D., McClure A. (2016). Insect Control Recommendations for Field Crops Soybeans.

[29] Venugopal P.D., Dively G., Herbert A., Malone S., Whalen J., Lamp W.O. (2016). Contrasting role of temperature in structuring regional patterns of invasive and native pestilential stink bugs. PLoS ONE..

[30] Zobel E.S., Hooks C., Dively G.P. (2016). Seasonal abundance, host suitability, and feeding injury of the brown marmorated stink bug, *Halyomorpha halys* (Heteroptera: Pentatomidae), in selected vegetables. J. Econ. Entomol..

[31] Blount J.L., Buntin G., Roberts P.M. (2016). Effect of planting date and maturity group on soybean yield response to injury by *Megacopta cribraria* (Hemiptera: Plataspidae). J. Econ. Entomol..

[32] NOAA (2020). Comparative climatic data. National Climatic Data Center, National Oceanic and Atmospheric Administration (NOAA).

